# Dietary Curcumin Promotes Gilthead Seabream Larvae Digestive Capacity and Modulates Oxidative Status

**DOI:** 10.3390/ani11061667

**Published:** 2021-06-03

**Authors:** Maria J. Xavier, Gian Marco Dardengo, Carmen Navarro-Guillén, André Lopes, Rita Colen, Luisa M. P. Valente, Luís E. C. Conceição, Sofia Engrola

**Affiliations:** 1 Centro Ciências do Mar (CCMAR), Universidade do Algarve, *Campus* de Gambelas, 8005-139 Faro, Portugal; mmxavier@ualg.pt (M.J.X.); gianmarco.dardengo@unicatt.it (G.M.D.); carmen.navarro@csic.es (C.N.-G.); acclopes@ualg.pt (A.L.); rcolen@ualg.pt (R.C.); 2 SPAROS Lda., Área Empresarial de Marim, Lote C, 8700-221 Olhão, Portugal; luisconceicao@sparos.pt; 3 Centro Interdisciplinar de Investigação Marinha e Ambiental (CIIMAR), Universidade do Porto, Terminal de Cruzeiros do Porto de Leixões, Avenida General Norton de Matos, S/N, 4450-208 Matosinhos, Portugal; lvalente@icbas.up.pt; 4 Instituto de Ciências Biomédicas de Abel Salazar (ICBAS), Universidade do Porto, Rua Jorge Viterbo Ferreira, 228, 4050-313 Porto, Portugal

**Keywords:** fish larvae, gilthead seabream, curcumin supplementation, growth performance, feeding incidence, gut fullness, digestive capacity, antioxidant status

## Abstract

**Simple Summary:**

The production of marine fish larvae is still recognized to have high mortality rates, so to achieve a more sustainable and competitive aquaculture industry it is essential to develop high-quality larvae. Therefore, this work evaluates if dietary supplementation of natural antioxidant curcumin enhances fish larvae robustness, hence improving growth performance and health status of the farmed fish. For that, two doses of curcumin were assessed and showed that curcumin can modulate larvae condition, digestive capacity, and antioxidant status throughout development. These results bring new perceptions of the effects of dietary curcumin in marine fish larvae that until now have not been studied. Moreover, this data suggested that curcumin can be a suitable feed additive contributing to the development and optimization of microdiets for fish larvae.

**Abstract:**

The larval stage is highly prone to stress due to the ontogenetic and metabolic alterations occurring in fish. Curcumin inclusion in diets has been shown to improve growth by modulating oxidative status, immune response, and/or feed digestibility in several fish species. The aim of the present work was to assess if dietary curcumin could promote marine fish larvae digestive maturation and improve robustness. Gilthead seabream larvae were fed a diet supplemented with curcumin at dose of 0 (CTRL), 1.5 (LOW), or 3.0 g/Kg feed for 27 days. From 4 to 24 days after hatching (DAH), no differences were observed in growth performance. At the end of the experiment (31 DAH) LOW larvae had a better condition factor than CTRL fish. Moreover, HIGH larvae showed higher trypsin and chymotrypsin activity when compared to CTRL fish. LOW and HIGH larvae were able to maintain the mitochondrial reactive oxygen species production during development, in contrast to CTRL larvae. In conclusion, curcumin supplementation seems to promote larvae digestive capacity and modulate the oxidative status during ontogeny. Furthermore, the present results provide new insights on the impacts of dietary antioxidants on marine larvae development and a possible improvement of robustness in the short and long term.

## 1. Introduction

Improving larvae quality in commercial hatcheries is critical to respond to a progressively higher demand of sustainable aquaculture in regard to fish species production. Thus, major advances have been made in the knowledge of marine larvae ontogeny [1,2], the development of formulated starter feeds [3,4], and feeding regimes [5,6]. However, marine fish hatcheries still face some challenges during early stages of development of most produced species. Most marine larvae present a low digestive capacity, with reduced acceptability and digestion efficiency for microdiets, and are very vulnerable and highly prone to stress, having strict requirements for biotic and abiotic conditions to survive [7,8,9]. Altricial marine fish larvae hatch from small and mostly pelagic eggs, and at first feeding they lack a fully developed digestive tract, typically showing a non-functional stomach and low digestive enzymatic activity [10,11]. In fact, most marine teleosts exhibit a drastic metamorphosis that involves the loss of embryonic features, undergoing several morphologic and metabolic changes to achieve the juvenile and adult form [12]. The timing of complete development of the gastrointestinal tract and organs related to prey capture can vary by weeks to months after first feeding, depending on the fish species. Moreover, with growth rates that can exceed 30% a day, larval stages present a remarkable growth potential [13,14]. These periods present a high energy and oxygen uptake demand, and thus may lead to higher susceptibility to oxidative stress. Indeed, metamorphosis and weaning are normally considered the most stressful moments for marine larvae production and are associated with high mortalities [6,15,16,17].

Production of reactive oxygen species (ROS) is inherent to cellular metabolism. Mitochondria are the major source of ROS production, as a small amount of electrons normally leak from mitochondrial electron transport system and leads to partial reduction of oxygen to form superoxide radicals [18]. Depending on the levels of ROS, they may induce a variety of responses in the cell. In moderate concentrations, ROS can act as signaling molecules of different vital cellular functions; however, in higher amounts they can cause oxidative stress by damaging a variety of biomolecules. Within the cell, a complex antioxidant system is composed by endogenous and exogenous molecules capable of counteracting and preventing the adverse effects of ROS. The maintenance of a redox balance between the production of ROS and the antioxidant defenses is essential to the normal function of the cells [19]. Thereby, it is critical to strengthen the larvae antioxidant status, contributing to better health and supporting fast growth.

The use of plant extracts as dietary supplements to promote health and growth performance in aquaculture production is gaining attention [20]. Curcumin, a pigment extract from the rhizome of turmeric (*Curcuma longa*), has been widely used for centuries in Southeast Asia in Ayurvedic medicine and consumed as a spice, flavoring agent, food preservative, and colorant [21]. Curcumin has been reported to perform a number of biological properties, like anti-inflammatory, antioxidant [22], and immunostimulant activities [23]. The multitude of beneficial effects of curcumin results from its ability to modulate a variety of enzymatic activities and expression of genes in farmed fish [24]. A high range of dietary curcumin supplementations (0.005–4%) increased growth performance and enhanced endogenous antioxidant defenses preventing lipid peroxidation in different fish species, such as in juveniles of Nile tilapia (*Oreochromis niloticus*) [25,26], common and grass carp (*Cyprinus carpio* and *Ctenopharyngodon Idella*) [27,28] and rainbow trout (*Oncorhynchus mykiss*) [29]. In different stress challenges, the use of curcumin at 0.005–0.02% inclusion in the diet for Nile tilapia juveniles resulted in an improvement in survival after an infection with *Aerossomas hydrophila* [26,30] and *Streptococcus iniae* [25]. Moreover, dietary curcumin has been shown to modulate growth performance and feed utilization through gastroprotective effects. In juveniles of crucian carp (*Carassius auratus*), the addition of 0.5% of curcumin to the diet improved growth performance and increased the digestive and absorptive ability; such effects were achieved through an enhancement of the expression of genes and activity of digestive enzymes such as trypsin and lipase in the hepatopancreas and intestine, and by an increase of the intestinal antioxidant capacity [31]. In climbing perch juveniles (*Anabas testudineus*) the inclusion of curcumin at 0.5 and 1% in the diet promoted a hypertrophy and hyper-activity of hepatopancreas, corroborating the effects of this antioxidant in promoting digestion and feed efficiency [32]. The use of this supplement in diets for early life stages of fish are still scarce, however recent studies have shown promising results with curcumin supplementation as promoter of growth through an upregulation of myogenic regulatory factors and by an improvement in the oxidative status of Senegalese sole postlarvae [33,34].

Gilthead seabream (*Sparus aurata*) is the most farmed fish species in the Mediterranean Sea [35], but the still low survival (around 25–30% in industrial hatcheries) and highly variable growth rates at the end of the first month of development are important constraints [36,37,38]. Therefore, the objective of the present study was to assess if the supplementation of curcumin during an early co-feeding regime at mouth-opening could enhance the robustness of gilthead seabream larvae through the improvement of digestive capacity and antioxidant status, therefore improving survival and growth rate in this critical early stage of development.

## 2. Materials and Methods

### 2.1. Husbandry and Experimental Set-up

Gilthead seabream larvae of 4 days after hatching (DAH) were supplied by the Laboratory of Marine Cultures at the University of Marine and Environmental Sciences (Cádiz, Spain) with an initial dry weight of 0.026 ± 0.007 mg larva^−1^ and transferred to Ramalhete Marine Station (CCMAR/Universidade do Algarve, Faro, Portugal).

Larvae were distributed in 9 cylindro-conical tanks (100 L) in a semi-closed recirculation system with an initial density of 284 larvae l^−1^ (28,400 larvae/tank). The 3 dietary treatments were randomly assigned to the 9 tanks, and treatments were tested in triplicates. The experimental period lasted for 27 days. The experimental rearing system was equipped with a mechanical filter, a submerged biological filter, a protein skimmer, and a UV sterilizer. Photoperiod was 10 h light, followed by 14 h dark. A daily monitoring of environmental parameters (mean ± SD; temperature 19.2 ± 0.02 °C, salinity 36.3 ± 0.6 psu and dissolved oxygen in water 93.8 ± 0.4% of saturation) and larval mortality was performed; the rearing tanks were cleaned regularly to preserve water quality.

### 2.2. Experimental Diets and Feeding Protocol

Three microdiets (CTRL, LOW, HIGH) were produced by SPAROS Lda. (Olhão, Portugal) to be isonitrogenous and isoenergetic. A commercial diet (WINFast, SPAROS Lda., Portugal) was used as control (CTRL), and in addition, the other two experimental diets were prepared by supplementing the CTRL diet with two levels of curcumin: LOW—curcumin supplementation at 1.5 g/kg feed and HIGH—curcumin supplementation at 3.0 g/kg feed. The curcumin (95.34 % purity) used in the supplemented diets was provided by Denk Ingredients (Munich, Germany). The selected levels of dietary curcumin were based on previous work on Senegalese sole postlarvae [33]. According to manufacturer’s data, these diets contained ingredients such as squid meal, wheat gluten, fish meal, crustacean meal, fish protein hydrolysate, gelatin, fish oil, lecithin, and a micronutrient premix comprising vitamins, minerals, and other additives. The diets were prepared by mixing a powder fraction of the control diet with the correspondent curcumin dosage. The powder mixture was subsequently humidified and agglomerated by low-shear extrusion (<60 °C). Upon extrusion, diets were dried in a convection oven (OP 750-UF, LTE Scientifics, Greenfield, UK) for 4 h at 60 °C, being subsequently crumbled (Neuero Farm, Melle, Germany) and sieved to desired size ranges. Proximal composition was identical for all 3 diets, with 63% WM crude protein, 17% WM crude fat, and 21.5 MJ kg^−1^ gross energy. These diets only changed in the supplementation with curcumin, and this supplementation did not exceed 1% of the diets.

Larvae from all treatments were fed according to a feeding plan based on rotifers (*Brachionus plicatilis*) enriched with DHA protein Selco (Inve, Dendermonde, Belgium), *Artemia* nauplii (Inve, Dendermonde, Belgium), and the experimental inert diets (Table 1). The amount of feed offered, co-feeding intervals, and inert diet size were the same for all treatments and dependent on larval age according to the following protocol: at mouth opening (4 DAH) larvae were fed on rotifers in co-feeding with inert diet. Rotifers were initially increased from 12 rotifers mL^−1^ to 16 rots mL^−1^ and then progressively reduced until 13 DAH. *Artemia* nauplii was introduced at an initial density of 0.3 AF mL^−1^ at 10 DAH and then gradually reduced until 0.15 AF mL^−1^ at 24 DAH (weaning). Inert diets were offered from 4 DAH to the end (31 DAH). The diets were initially supplied at a size of 100–200 μm from 4 to 11 DAH, then at an equal mixture (50/50) of 100–200 μm/200–400 from 12 to 24 DAH, and finally they were supplied at a size of 200–400 μm until the end of the experimental period (31 DAH). The total daily amount of inert diet distributed to each tank was divided in five meals while the total amount of live preys was initially divided in three meals (4–9 DAH) and later reduced to two times per day (10–23 DAH). Larval rearing system was based on green water technique using *Nannochloropsis oculata* (4–23 DAH).

### 2.3. Growth and Survival

Survival rate (%) for each treatment was determined at the end of the experiment (31 DAH) by direct counting of individuals, by the formula:

(1) Survival (%)=(final fish number ÷ initial fish number)×100

Growth performance at each sampling point was assessed by individual dry weight (DW) and total length (TL) measurements (*n* = 15 per replicate, except for larvae at 4 DAH where a pool of 30 larvae per replicate was considered). Dry weight measurements were obtained after freeze-drying the samples using a high precision microbalance (± 0.001 mg; MSA36S-000-DH, Sartorius, Germany); previously, larvae were washed twice in distilled water and snap-frozen in liquid nitrogen. Total length was performed using the Leica Application Suite LAS (Leica Microsystems, Wetzlar, Germany) for digital image analysis. The same larvae were used for dry weight and total length in order to calculate larval condition factor (K). Based on those parameters the condition factor was calculated according to Fulton’s condition factor formula:

(2) $$K\;=\;\mathrm{final}\;\mathrm{body}\;\mathrm{weight}\;\left(\mathrm{mg}\right)\;÷\;{\left[\mathrm{final}\;\mathrm{body}\;\mathrm{length}\;\left(\mathrm{mm}\right)\right]}^{3}$$

Daily individual growth was evaluated by the relative growth rate (RGR, % day^−1^) determination following the formula [39]:

(3) $$\mathrm{RGR}\;=\left(e^{g}-1\right)\times 100,\;\mathrm{where}\;g\;=⎣(\ln_{\mathrm{final}\;\mathrm{weight}\;}-{\;\ln}_{\mathrm{initial}\;\mathrm{weight}})\;÷\;\mathrm{time}⎦$$

### 2.4. Feeding Incidence and Gut Fullness

To evaluate the feeding incidence (absence/presence of feed in the larva gut) 10 individual larva per replicate (*n* = 30 per treatment) were sampled at 5, 6, 8, 12, 16, 20, 23, and 28 DAH, always at 2:00 p.m. to ensure the same feeding status between sampling days. For gut content estimation, gut fullness level was examined by image analysis based on the techniques previously described [40,41]. Each larva was photographed under a microscope connected to the Leica Application Suite (LAS) for digital image analysis. The level of gut fullness was determined measuring the pigmented area within the digestive tract. Gut content was normalized for larva size using the ratio between the fullness area and the total length of each larva. For feeding incidence estimation, per replicate tank and sampling point, larva with gut fullness lower than 10% with respect to the maximum recorded was considered empty. The data analysis was performed using ImageJ software (National Institute of Health, Bethesda, MD, USA) [42]. 

### 2.5. Gut Maturation

Gut maturation was evaluated through the analysis of larvae digestive enzyme activity, such as trypsin, chymotrypsin, aminopeptidase-N, amylase, lipase, and alkaline phosphatase. Larvae were sampled at three developmental ages: 10 DAH (*n* = 3 pools of 4 larvae per replicate), 24 DAH (*n* = 5 pools of 3 larvae per replicate), and 31 DAH (*n* = 2 pools of 2–3 larvae per replicate). Samples were freeze-dried and manually homogenized in 250 μL (10 DAH), 230 μL (24 DAH), and 350 μL (31 DAH) of distilled water. The homogenate was centrifuged for 5 min at 12,500× *g* at 4 °C to remove the tissue, and the enzymatic extract (supernatant) was used for the analysis. All samples were kept in ice during the process described above to avoid enzyme denaturation and/or damage. Enzyme extracts were kept at −20 °C until further analysis. For protease activity measurement, trypsin, chymotrypsin, and aminopeptidase-N, the fluorogenic substrates Boc-Gln-Ala-Arg-7- methylcoumarin hydrochloride (BOC, Sigma-Aldrich, St. Louis, MO, USA; B4153), N-Succinyl-Ala-Ala-Phe-7-amido-4-methylcoumarin (Sigma-Aldrich, St. Louis, MO, USA; S8758), and Nα-Benzoyl-L-arginine-7-amido-4-methylcoumarin hydrochloride (Sigma-Aldrich, St. Louis, MO, USA; B7260) respectively were diluted in dimethyl sulfoxide (DMSO) to a final concentration of 20 μM. For analysis, 5 μL of substrate, 190 μL of 50 mM Tris + 10 mM CaCl_2_ buffer (pH 8.5, without CaCl_2_ for aminopeptidase), and 15 μL of the larval homogenate were added to the microplate [43,44]. Fluorescence was measured at 355 nm (excitation) and 460 nm (emission). Ultra Amylase Assay Kit (E33651) from Molecular Probes was used for amylase analysis. This kit contains a starch derivate labeled with a fluorophore dye as substrate. This substrate was diluted in substrate solvent (sodium acetate; pH 4.0) and reaction buffer (0.5 M MOPS; pH 6.9) to a final concentration of 200 μg/mL. For analysis, 50 μL of the substrate solution and 15 μL of the larvae extract were added to the microplate. Fluorescence was measured at 485 nm (excitation) and 538 nm (emission). Lipase activities were assayed using 4-methylumbelliferyl butyrate (Sigma-Aldrich, St. Louis, MO, USA; 19362), and 4-methylumbelliferyl oleate (Sigma-Aldrich, St. Louis, MO, USA; 75164) as substrates for 4-C and 18-C like lipases, respectively. Substrates were dissolved in phosphate buffer (pH 7.0) to a final concentration of 0.4 mM, modified method from Rotlland et al., 2008 [44], aliquoted and stored at −20 °C. Larvae homogenate (15 μL) was added to the microplate and mixed with 250 μL of 0.4 mM substrate for the analysis. Fluorescence was measured at 355 nm (excitation) and 460 nm (emission). For alkaline phosphatase analysis, the substrate used was 4-methylumbelliferyl phosphate disodium salt, (MUP, Sigma-Aldrich, St. Louis, MO, USA; M8168). A 1 mmol/l stock solution of MUP was prepared by dissolving the substrate in borate buffer (pH 8). The enzymatic extract (15 μL) was added to the microplate and mixed with 100 μL of substrate for the analysis (modified from Fernley et al., 1965 [45]). Fluorescence was measured at 360 nm (excitation) and 440 nm (emission). All enzyme activities were expressed as RFU (Relative Fluorescence Units) per mg larva dry weight.

### 2.6. Antioxidant Status

The antioxidant status of the larvae was assessed by measuring the following oxidative stress biomarkers: total glutathione (GSH), total antioxidant capacity (TAC), protein carbonylation (PC), and mitochondrial reactive oxygen species production (mtROS). Samples were taken at 10 DAH (*n* = 1 pool of 50 larvae, *n* = 3/treatment), at 24 DAH (*n* = 1 pool of 30 larvae, *n* = 3/treatment), and at 31 DAH (*n* = 1 pool of 20 larvae, *n* = 3/treatment). Larvae were washed twice in distilled water and then snap-frozen in liquid nitrogen and stored at −80 °C until further analysis.

#### 2.6.1. Sample Preparation for Biomarker Analysis

For the analysis of GSH, TAC, and PC, larvae samples were homogenized using a TissueLyser (Star-Beater, VWR, Radnor, PA, USA) in 1200 μL of ultra-pure water. The supernatant (700 μL) was diluted in 0.2 M K-phosphate buffer (pH 7.4, vol. 1:1), and centrifuged for 10 min at 10,000× *g* at 4 °C. The post-mitochondrial supernatant (PMS) was divided into aliquots for GSH, TAC, and PC analysis.

To determine mtROS production, larvae samples were homogenized using a TissueLyser (Star-Beater, VWR, Radnor, PA, USA) in 1 mL of buffer containing 225 mM manitol, 75 mM sucrose, 1 mM EDTA, and 4 mM HEPES (pH 7.2) following the protocol described by Da Silva et al., 2015 [46]. The homogenate was centrifugated for 10 min at 1200 g and 4 °C. The supernatant was carefully removed and centrifugated again for 10 min, at 16,500 g and 4 °C. The pellet was re-suspended in a buffer containing 250 mM sucrose and 5 mM HEPES (pH 7.2). The volume of buffer utilized was at 10 DAH (500 μL), at 24 DAH (650 μL) and at 31 DAH (750 μL).

The samples were kept on ice during the assay and then maintained in −80 °C until further analysis. All biomarker determinations were performed spectrophotometrically, in 96-well flat bottom microplates, with a temperature-controlled microplate reader (Synergy 4 BioTek, Winooski, VT, USA). Protein concentration of PMS and mtROS was determined according to the Bradford method [47], using bovine γ-globulin as a standard.

#### 2.6.2. Oxidative Status Biomarker Measurements

Total glutathione content (GSH) was determined at 412 nm using a recycling reaction of reduced glutathione with 5,5′-dithiobis-(2-nitrobenzoic acid) (DTNB) in the presence of glutathione reductase excess [48,49]. GSH content was calculated as the rate of TNB^2−^ formation with an extinction coefficient of DTNB chromophore formed, ε = 14.1 × 10^3^M^−1^cm^−1^ [48,50]. Results were expressed in mmol GSH per mg protein. Total antioxidant capacity (TAC) was assessed following the protocol described by Erel, 2004 [51], using colored 2,2-azino-bis-(3-ethylbenzothiazoline-6-sulfonic acid) radical cation (ABTS^+^). This method is based on the colorless molecule ABTS, which is oxidized to a characteristic blue-green ABTS^+^. This change in color was measured as a change in absorbance at 660 nm and the assay was calibrated with Trolox. Results were expressed in mmol Trolox equivalent per mg protein. Protein carbonylation (PC) was measured based on the reaction of 2,4-dinitrophenylhydrazine (DNPH) with carbonyl groups, according to the DNPH alkaline method [52]. The amount of carbonyl groups was quantified spectrophotometrically at 450 nm at room temperature against a blank (22,308 mM^−1^cm^−1^ extinction coefficient). Results were expressed in nmol carbonyl per mg protein. The mitochondrial reactive oxygen species (mtROS) production was assessed by the dihydrodichloro-fluorescein diacetate –H(2)DCF-DA [46,53]. This dye is non-fluorescent when chemically reduced, but after cellular oxidation and removal of acetate groups by cellular esterases it becomes fluorescent [54]. The mitochondrial suspension was incubated in the presence of DCFDA and fluorescence was monitored over 5 min, with excitation and emission wavelengths of 503 and 529 nm, respectively. Under the described conditions, the linear increment of fluorescence indicated the rate of ROS formation. Results are expressed as Relative Fluorescence Units (RFU) per mg mitochondrial protein.

### 2.7. Statistical Analysis

All data were tested for normality using a Kolmogorov–Smirnov (whenever *n* > 30) or Shapiro–Wilk (whenever *n* < 30) test and for homogeneity of variance using a Levene’s test using IBM SPSS Statistics v19 software (IBM, Armonk, NY, USA). Data were log transformed when required and percentages were arcsin (SRQRT) transformed prior analysis [55]. To assess the effects of the different dietary treatments in the ontogenetic development of the larvae a one-way ANOVA followed by a Tuckey’s post-hoc test was performed for the analyses of growth performance, feed incidence, gut maturation, digestive enzyme activity, and oxidative status for each treatment along larvae age. Comparisons between groups fed different diets were made using one-way ANOVA followed by a Tukey’s post-hoc test for growth performance, gut fullness, digestive enzyme activity, and oxidative status at each larvae age; for assessing larvae feed incidence a chi-square test was used at each larvae age. In addition, a linear regression was performed to estimate association between feeding incidence and larvae age. Significance levels were set at *p* < 0.05.

## 3. Results

### 3.1. Growth Performance

The dietary curcumin did not improve gilthead seabream growth performance (DW and TL) along larvae developmental stages (*p* > 0.05) (Table 2). At the end of the growth trial, dietary curcumin in the LOW diet was able to significantly improve the condition factor (K) when compared to larvae fed the non-supplemented CTRL diet (*p* = 0.016). All larvae presented similar relative growth rate (RGR) throughout the experiment (*p* > 0.05), between 8.0–8.5% mg DW.day^−1^. On average, survival rate was 1.88 ± 0.51% (*p* = 0.381).

### 3.2. Feeding Incidence

**Figure 1:**
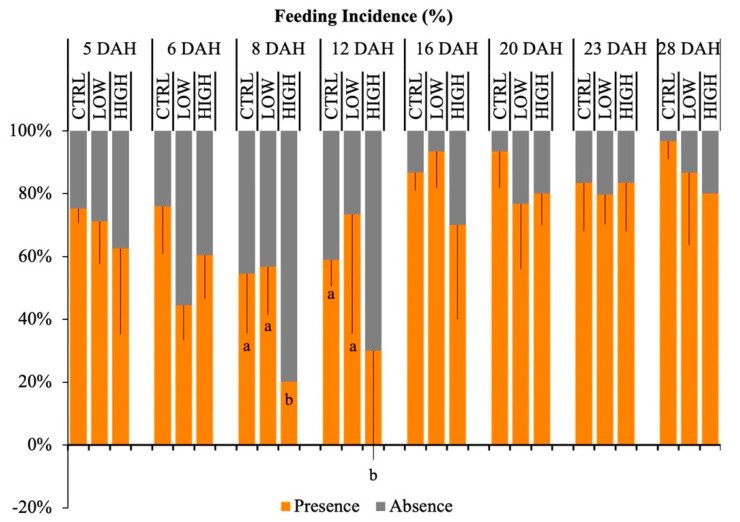
Feeding incidence (%) of gilthead seabream larvae throughout ontogeny (5, 6, 8, 12, 16, 20, 23, and 28 days after hatching, DAH) fed experimental diets (CTRL, LOW, and HIGH). Values are expressed as mean ± SD. Different letters indicate statistical differences between dietary treatments at the same larval age (a, b; *p* < 0.05, chi-square). Absence of letters indicates no statistical differences (*p* > 0.05).

**Figure 2:**
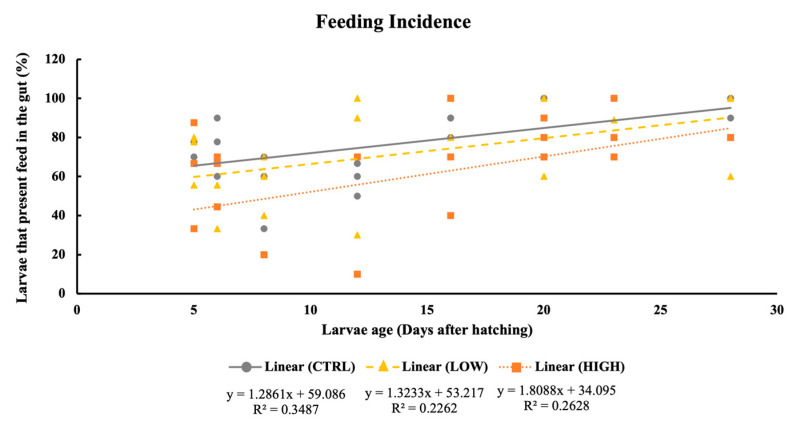
Linear regression between gilthead seabream larvae feeding incidence and age fed experimental diets (CTRL, LOW, and HIGH).

On average, more than 40% of the larvae presented feed in the gut throughout the experiment. A significant decrease in feeding incidence was registered in larvae fed the HIGH diet at 8 and 12 DAH when compared to the CTRL and LOW larvae (*p* < 0.05) (Figure 1). Regarding the ontogenetic development of feeding incidence within treatments, only in CTRL and HIGH were there differences observed (*p* < 0.05). Overall, feeding incidence increased with age in CTRL and HIGH larvae. In contrast, the feeding incidence in the LOW treatment was similar across larvae development (*p* = 0.151). All treatments showed a significant positive correlation between feeding incidence and larvae age (CTRL—*p* = 0.002; LOW—*p* = 0.019; HIGH—*p* = 0.010, Figure 2).

**Figure 3:**
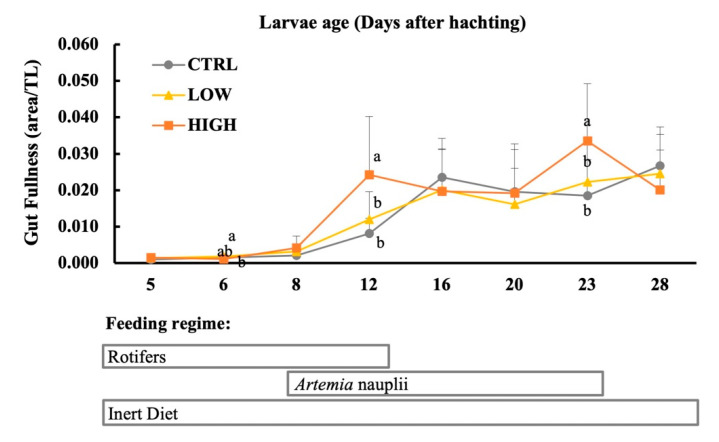
Gut fullness (area/TL) of gilthead seabream larvae throughout ontogeny fed different diets (CTRL, LOW, and HIGH). Values are expressed as mean ± SD. Different subscription letters indicate statistical differences between dietary treatments at the same age (a, b; *p* < 0.05, 1-way ANOVA). Absence of letters indicates no statistical differences (*p* > 0.05).

Gut fullness was the highest in the larvae fed HIGH diet in comparison to the remaining dietary treatments at 12 and 23 DAH (*p* < 0.05, Figure 3). Regarding the impact of development on gut fullness, all dietary treatments were able to stimulate shifts (*p* < 0.05). The larvae from CTRL showed the lowest gut content between 5 to 8 DAH and the highest from 16 DAH onwards (*p* < 0.005). Similarly, the gut content of the larvae fed the LOW diet showed the lowest values between 5 to 6 DAH and the highest levels from 16 DAH until the end of the trial. In contrast, in HIGH larvae the increment on gut fullness was observed earlier when compared with the other treatments, from 12 DAH onwards.

### 3.3. Digestive Enzymes

The HIGH dietary treatment was able to positively modulate trypsin and chymotrypsin activity levels in gilthead seabream larvae (Table 3). Both at 24 and 31 DAH, the larvae fed the HIGH diet showed the highest activity of trypsin yet only at the end of the growth trial was this difference significant compared to the CTRL (*p* = 0.002). Regarding the ontogenetic development of these enzyme activity levels, all larvae showed similar patterns of activity, with the lowest activity recorded at 24 DAH followed by a significant increase at 31 DAH (*p* < 0.005). Chymotrypsin activity was only detected in larvae 31 DAH; enzyme activity levels were higher in HIGH larvae compared to CTRL larvae (*p* = 0.004). Aminopeptidase activity was only detected in fish 31 DAH, with no differences between dietary treatments (*p* = 0.463). A higher activity level of 4C-like lipase at 10 DAH was observed in LOW larvae, compared to HIGH larvae (*p* = 0.001) (Table 3). However, at 24 DAH the activity levels of this enzyme were the lowest in the larvae fed curcumin (HIGH and LOW) (*p* = 0.001). At the end of the growth trial, the activity of 4C-like lipase was similar in all treatments (*p* = 0.537). Differences in patterns regarding the ontogenetic development of this enzyme activity levels were observed in the curcumin treatments (HIGH and LOW) compared to CTRL. All treatments showed the highest activity of 4C-like lipase at 10 DAH and a significant decrease at 24 DAH (*p* < 0.005). However, the HIGH and LOW treatments were able to significantly improve these activity levels at 31 DAH, in contrast to CTRL, which were able to maintain similar levels between 24–31 DAH. The activity of 18C-like lipase was only detected at 24 and 31 DAH larvae. At 24 DAH, CTRL larvae showed higher 18C-like lipase activity than LOW and HIGH larvae (*p* < 0.001). At the end of the experiment, a global decrease in the activity of this enzyme was observed for all the treatments, leveling activity levels between treatments (*p* = 0.966). Alkaline phosphatase activity was detected at 24 and 31 DAH larvae (Table 3). At 24 DAH, activity levels from CTRL fed fish were significantly higher than in HIGH larvae (*p* = 0.016). However, at 31 DAH, larvae from all treatments presented the same activity levels (*p* = 0.705). This seems to be explained by a significant modulation of curcumin treatments (LOW and HIGH) in the ontogenetic development of alkaline phosphatase activity (*p* < 0.005). The larvae from LOW and HIGH treatments showed a significant improvement in the activity levels of this enzyme from 24 to 31 DAH. In contrast, CTRL larvae showed no differences between the two ages (*p* = 0.055). Amylase activity was only detected in 24 and 31 DAH larvae, showing no differences between treatments and with an overall, decreasing pattern in the enzyme activity along larvae development (*p* < 0.05).

**Figure 4:**
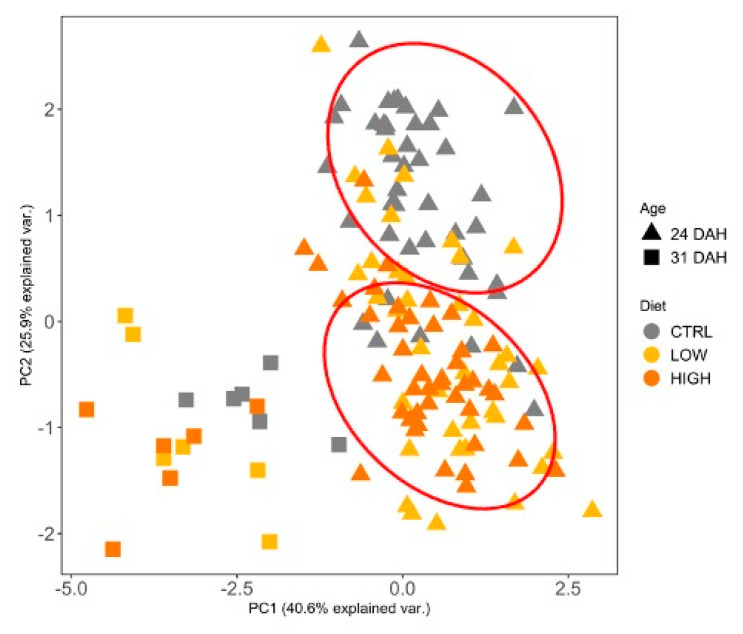
Principal component analysis (PCA) of the activity of several digestive enzymes (trypsin, 14C- and 18C-like lipase, alkaline phosphatase, and amylase) of gilthead seabream larvae at 24 and 31 DAH fed different diets (CTRL, LOW, and HIGH). Light grey, dark grey, and black dots represent CTRL, LOW, and HIGH, respectively; triangle and squares represent 24 and 31 DAH larvae; Red ellipses are merely indicative to facilitate the visualization of the major distances occurring between treatment CTRL and HIGH at 24 DAH.

Principal component analysis (PCA) was employed to explore the combined effects of variables on the dietary treatments at two stages of development (Figure 4). A score scatter plot was generated with the projection of the samples on the first two principal components (PCs) which accounted for 41% and 26% of the total variability of the data, respectively. The observation of the sample groupings in the score plots suggests a clear separation between larvae ages (24 and 31 DAH) along PC1 axis. Hence, the PC2 seems to explain the differences between CTRL and HIGH treatments at 24 DAH. The loading plots, which explained the weight of each variable on the PCs, suggest that trypsin (−0.3) and 14C- and 18C-like lipase (0.5; 0.8, respectively) were the main elements responsible for those differences in the PC2 axis.

### 3.4. Antioxidant Status

All the oxidative stress biomarkers (GSH, TAC, PC, and mtROS) were similar between dietary treatments throughout larvae development (*p* > 0.05). However, curcumin supplementation (LOW and HIGH diets) seems to differently modulate these physiological indicators across larvae ontogeny (Table 4). The content of GSH increased during larvae development in CTRL and HIGH treatments (*p* > 0.05). In contrast, no differences were observed in the content of this endogenous antioxidant along the ontogeny of larvae fed the LOW diet. The larval TAC was higher at 10 DAH and decreased in older larvae in CTRL treatment (*p* = 0.005). Similarly, in LOW treatment a higher TAC at 10 DAH was also observed, but it was only statistically different from 24 DAH larvae (*p* = 0.027). In contrast, no differences were recorded in TAC during larval development in the HIGH treatment (*p* = 0.051). The content of PC differed neither during larval development nor between treatments (*p* > 0.05). In CTRL larvae, mtROS production was significantly lower at 10 DAH compared to later ages (*p* < 0.001). In opposition, the mtROS formation did not change during development in larvae from curcumin treatments (*p* > 0.05).

**Figure 5:**
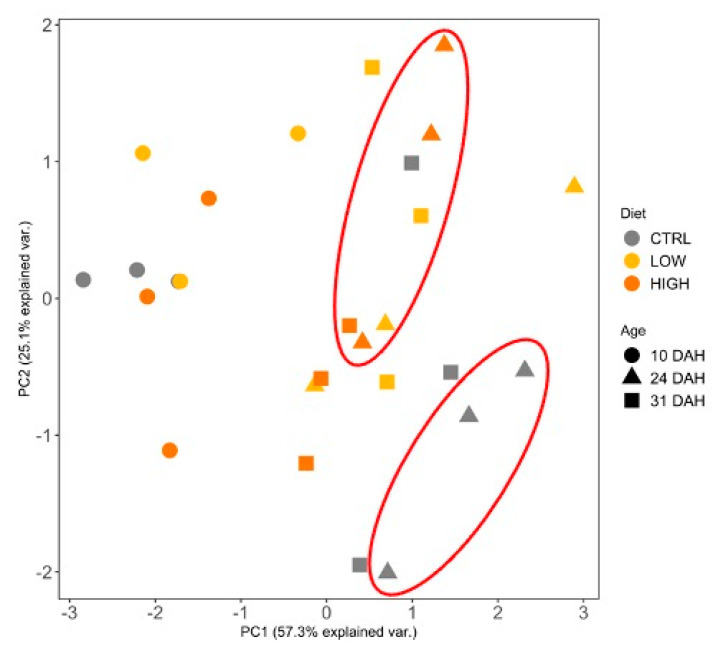
Principal component analysis (PCA) of biomarkers of oxidative status of gilthead seabream larvae throughout ontogeny fed different diets (CTRL, LOW, and HIGH). Light grey, dark grey, and black dots represent CTRL, LOW, and HIGH, respectively; circles, triangles, and squares represent 10, 24, and 31 DAH larvae. Red ellipses are merely indicative to facilitate the interpretation of the major distance between treatment CTRL and HIGH at 24 DAH.

PCA was performed to explore the combined effects of variables on dietary treatments at different larvae ages (Figure 5). PC1 explained 57% of the total variance observed in the score plots, showing a dissociation between larvae ages (10 DAH from 24 and 31 DAH). The PC1-loadings showed that GSH (0.6), TAS (−0.6), and mtROS (0.6) were the main contributors for the differences observed between ages. The PC2 explained 25% of the variation and seems to explain the differences between CTRL and HIGH treatments at 24 DAH.

## 4. Discussion

In this work the inclusion of curcumin in microdiets (LOW and HIGH) for gilthead seabream larvae showed no effects in larvae growth performance throughout ontogeny. However, the condition factor (K) at the end of the growth trial (31 DAH) was higher in LOW larvae. This index usually relates to the well-being and nutritional status of the fish [56,57,58]. Previous studies in freshwater species [25,26,27,28,29,30,31] and more recently in marine species [33,34] have shown a growth-promoting effect of dietary curcumin. However, these promising results are highly dependent on the dose and the species. Therefore, our results seem to suggest that curcumin may modulate growth of gilthead seabream larvae, but the doses of inclusion might need to be adjusted. The results for DW and TL were slightly lower compared to previous observations reported in the literature for gilthead seabream at the same age [59,60,61]. These differences might be explained by the more extreme feeding plan used in the present study, with co-feeding from mouth-opening with substantial live-feed replacement. This contrasts with the traditional feeding plans observed in the other studies, where live-feed was supplied at higher amounts and for a longer period. Moreover, survival rate at the end of the experiment was 4–5-fold lower compared to previous experiments with gilthead seabream at mouth opening, reared at similar conditions [38,59]. The larvae survival when weaning is started at mouth opening is usually lower when compared to a late weaning in older larvae (16–22 DAH), that can vary between 11–56% [60,61,62]. However, it was similar among treatments, implying that curcumin supplementation had no effect on the observed survival rates.

The feeding success in early larvae stage depends on a series of factors such as locomotion capacity, development of sensorial organs, and mouth size [2]. The use of several plant extracts (*Zingiber officinalis*, *Allium sativum, Andrographis paniculata, Cissus quadrangularis,* and *Eclipta alba*) showed to improve feed intake in early larval stages of different aquatic species (*Penaeus monodon*, *Clarias gariepinus, and Macrobrachium rosenbergii*), by acting as feed attractants [63,64,65]. In the present study, in order to obtain a qualitative analysis of the larval feed intake, feeding incidence was assessed throughout different sampling points (5–28 DAH) during the experimental period. Overall, the LOW larvae showed similar feed ingestion throughout ontogeny, whereas CTRL and HIGH larvae showed a more unstable feeding incidence. Furthermore, the HIGH larvae had the lowest consumptions (below 40%) compared to the other treatments at 8 and 12 DAH. All treatments showed a positive correlation between feeding incidence and larvae age. However, the HIGH larvae was correlated with the higher slope, which might indicate that these larvae presented a slower adaptation to the inert diet (with less feed ingestion at early ages when compared to the other treatments). Nevertheless, the results of gut content were significantly higher in HIGH larvae than in the other treatments at 12 and 23 DAH. In fact, the HIGH diet enhanced gut fullness in the larvae during the experiment and also promoted an early increase of the gut content (from 8 DAH). The effects of the two curcumin-supplemented diets (LOW and HIGH) in larvae feeding incidence and gut fullness were distinct. The HIGH diet decreased larval feeding incidence in some points of larval development, which might indicate that a higher inclusion of curcumin might decrease feed palatability. However, a higher gut content was further observed in the same larvae, suggesting that these larvae might present a slower adaptation to the diet, explaining the erratic feeding behavior. Nevertheless, after microdiet adaptation these larvae presented higher feed consumption compared to larvae from remaining treatments. In other studies, several plant extracts were described to have feeding attractability properties through the stimulation of the olfactory system, like in juveniles of oriental weatherfish (*Misgurnus anguillicaudatus*) and yellowtail (*Seriola quinqueradiata*) [66]. Moreover, dietary supplementation of plant extracts may stimulate the appetite and enhance feed consumption by inducing the production of digestive enzymes, then stimulating transit time [67,68].

The use of curcumin in weaning diets as promoter of gut maturation was already reported in broiler chicken [69]. In fish, the only studies concerning the effect of this supplement as a digestive promoter were only reported in juvenile or adults and not in early stages, contrarily to observations in higher vertebrate species. The present study showed that, at the end of the growth trial, the HIGH diet was able to improve the activity of trypsin and chymotrypsin in larvae when compared to the CTRL fish. These two pancreatic serine proteases have a complementary effect in the protein digestion by cleaving different peptide active chains. Moreover, trypsin also regulates the activation of its own precursor and other pancreatic proteases in the gut lumen, being recognized as the most important proteolytic enzyme in the early stage of marine fish larvae [2,70]. Therefore, the current results might indicate that a high dietary inclusion level of curcumin can improve protein digestibility in early larval stages, which might lead to an enhancement of protein accretion and ultimately increased growth performance in the long-term. The fact that the promoting effect of the HIGH diet in the larvae digestive capacity did not reflect in a higher growth at the end of the growth trial might be related to the irregular feeding incidence observed throughout ontogeny. Moreover, the observed effects of plant extract supplementation in fish have been shown to be dose dependent. In fact, the inclusion of Chinese herbal medicines mixture at different doses (5, 10, 15, 20, 25 g/kg) in the diet of Japanese seabass (*Lateolabrax japonicus*) showed that 20 g/kg doses improve growth and were correlated with increase in the activity of trypsin and lipase [71]. However, the higher dose, that was 1.25-fold higher than 20 g/kg, did not promote growth even though it did promote lipase activity. Therefore, the HIGH diet might need a fine tuning in order to have stable feed incidence and potentiate the effect of curcumin in the digestive capacity of this larvae and ultimately improve growth performance. Likewise, other studies used curcumin supplementation to promote the activity and transcription of trypsin in the intestine and hepatopancreas of crucian carp [31] and increased protease activity in tilapia [72]. In contrast, our data reports a decrease in the activity of 4C- and 18C-like lipases at 24 DAH in the larvae fed diets with curcumin inclusion (LOW and HIGH). However, larvae fed the LOW and HIGH diets were able to increase lipase activity to levels similar to the CTRL, at the end of the experiment. Thus, the reduction in the activity of lipases at 24 DAH apparently did not have an impact on growth performance of the fish fed curcumin during the experiment. This contrasts with the well-known effect of curcumin as promoter of lipid digestion [73]. In fact, in two studies in tilapia and crucian carp, higher lipase activity was reported with dietary curcumin supplementation [31,72]. Moreover, it was visible in the present study that the activity of alkaline phosphatase (AP) was lower in larvae fed the LOW diet at 24 DAH in comparison to CTRL fish. However, at 31 DAH the larvae fed the LOW diet showed the sharpest increase of this enzyme activity, reaching similar values to the remaining dietary treatments. Both larvae fed the supplemented diets were able to significantly modulate the activity of AP compared to CTRL larvae. The increase of this brush border membrane enzyme is usually correlated to the maturation of fish larvae enterocytes. In fact, in European seabass the maturation of digestive tract was accomplished by a decrease in amylase and an enhancement of trypsin and brush border membrane enzymes [74]. The activity of intestinal alkaline phosphatase was reported to increase with the inclusion of curcumin in diets for crucian carp [31] and Nile tilapia [25]. Therefore, this might corroborate the higher peak observed in the LOW and HIGH larvae in this work.

The use of curcumin has been shown to improve oxidative status in different fish species due to the recognized antioxidant properties. In this work, the dietary supplementation of curcumin in gilthead seabream larvae did not improve larvae endogenous antioxidant defenses nor decreased oxidative damage compared to the CTRL larvae in any of the developmental stages studied. Nevertheless, the dynamics of oxidative status during ontogeny changed between dietary treatments. Similarly, a study performed in postlarvae of Senegalese sole (*Solea senegalensis*) fed dietary curcumin showed no differences in the content of GST, PC and TAS compared to control diets. However, sole postlarvae were able to improve oxidative status by a decrease in the stress-related biomarkers [34]. In fact, in this work a differential modulation in fish antioxidant defenses was observed along larvae ontogeny, when fed the supplemented diets. The TAC content observed in larvae fed the HIGH diet was maintained across ontogeny in contrast with the decrease observed in the other dietary treatments at later ages, which might indicate that a higher dose of this antioxidant can ameliorate the oxidative status throughout larvae development. Moreover, the larvae from LOW treatment did not significantly increase the content of GSH along larvae ontogeny. Thus, the steady state of the biomarkers in LOW larvae could reflect an improvement in oxidative status, compared to the other dietary treatments, in which the fluctuation of GSH might be the result of coping with the high oxidative stress at early ages of development. The production of mitochondrial ROS, the primary source of endogenous ROS, was significantly increased along larvae development in the larvae fed the CTRL diet compared to the LOW and HIGH larvae, suggesting a possible mitigation of the endogenous production of mtROS in the larvae fed curcumin treatments. Other reports showed that curcumin supplementation can significantly improve TAC in Nile tilapia [25] and GSH content in crucian carp [31] and tilapia [26]. These results might corroborate that curcumin is able to modulate oxidative status of fish but still need further research to understand the pathways in which curcumin acts.

## 5. Conclusions

Overall, the present results show that a higher inclusion of curcumin (HIGH diet) improved larvae digestive capacity and feed intake. Moreover, a lower dose (LOW diet) enhanced larvae condition and contributed to a more consistent feeding incidence throughout larvae development. Nevertheless, the two experimental diets were able to positively modulate the digestive capacity of gilthead larvae, in particular for protein. The two curcumin-supplemented diets also seem to have modulated positively the oxidative status during early ontogeny. Therefore, a fine-tuning of a microdiet with a high curcumin content might improve palatability and faster acceptability of the diet, hence promoting larvae feed intake and ultimately growth performance and larval robustness. The present results bring new insights on the effects of dietary curcumin in growth performance, feeding incidence, digestive functionality, and oxidative status of fish larvae throughout ontogeny.

## Figures and Tables

**Table 1:** Feeding regime of gilthead seabream larva throughout the experimental period (4 to 31 days after hatching, DAH).

	Feeding Plan			
Age (DAH)	Rotifers	*Artemia* Nauplii	*Artemia* Metanauplii	Inert Diet
3	12			500
4	16			500
5	16			500
6	16			500
7	12			500
8	13			500
9	14			500
10	14	0.3		500
11	7	0.3		500
12	7	0.3		500
13	4	0.3		500
14		0.3		750
15		0.2		1000
16		0.2		1000
17		0.2		1000
18		0.2		1200
19		0.2		1200
20		0.2		1200
21		0.2		1500
22		0.2		1500
23		0.2		1600
24				2000
25				2000
26				2100
27				2200
28				2300
29				2400
30				2600
31				2800

Rotifers are expressed as ‘number of rotifers/mL/day’, Artemia are expressed as ‘number of artemia/mL/day’ and inert diet daily rations are expressed as ‘mg/tank/day’.

**Table 2:** Growth performance indicators of gilthead seabream larvae throughout the experimental period fed the different diets (CTRL, LOW, and HIGH).

		Treatments			1-Way Anova
		CTRL	LOW	HIGH	*p* Value
DW (mg)					
	4 DAH	0.026 ± 0.007			
	10 DAH	0.020 ± 0.005	0.020 ± 0.006	0.019 ± 0.004	0.059
	24 DAH	0.143 ± 0.061	0.140 ± 0.068	0.142 ± 0.059	0.973
	31 DAH	0.207 ± 0.082	0.243 ± 0.102	0.221 ± 0.089	0.238
TL (mm)					
	10 DAH	3.544 ± 0.264	3.593 ± 0.179	3.574 ± 0.159	0.529
	24 DAH	5.504 ± 0.691	5.434 ± 0.645	5.525 ± 0.659	0.795
	31 DAH	5.909 ± 0.617	6.029 ± 0.566	5.943 ± 0.675	0.697
K					
	10 DAH	0.219 ± 0.059	0.216 ± 0.074	0.202 ± 0.028	0.583
	24 DAH	0.421 ± 0.067	0.393 ± 0.073	0.402 ± 0.062	0.183
	31 DAH	0.476 ± 0.081 ^b^	0.538 ± 0.115 ^a^	0.517 ± 0.099 ^ab^	0.016
RGR (% day^−1^)					
	10–24 DAH	14.904 ± 1.674	14.910 ± 2.232	15.977 ± 1.033	0.694
	24–31 DAH	5.807 ± 4.739	7.720 ± 4.123	5.565 ± 1.98	0.784
	4–31 DAH	8.069 ± 0.501	8.509 ± 0.809	8.020 ± 0.896	0.698
Survival (%)	4–31 DAH	2.069 ± 0.536	2.045 ± 0.691	1.539 ± 0.104	0.381

Values are expressed as mean ± SD. Different subscription letters (a, b) indicate differences between larvae from different dietary treatments at the same age (*p* < 0.05). Absence of letters indicates no statistical differences (*p* > 0.05). DW, dry weight; TL, total length; K, condition factor; RGR, relative growth weight; DAH, days after hatching.

**Table 3:** Activity of several digestive enzymes in gilthead seabream larvae throughout ontogeny fed different diets (CTRL, LOW, and HIGH).

		Treatments			1-Way Anova
		CTRL	LOW	HIGH	*p* Value
Trypsin (RFU/ mg protein)					
	10 DAH	30,459.3 ± 13,867.0	31,570.1 ± 14,050.5	54,140.9 ± 43,501.8	0.158
	24 DAH	5337.5 ± 2949.4 ^ab^	4053.2 ± 2962.1 ^b^	6722.3 ± 3916.7 ^a^	0.003
	31 DAH	58,992.9 ± 12,290.3 ^b^	78,515.8 ± 12,833.5 ^ab^	102,968.8 ± 24,976.4 ^b^	0.002
Chymotrypsin (RFU/mg protein)					
	10 DAH	n.d	n.d	n.d	
	24 DAH	n.d	n.d	n.d	
	31 DAH	55,218.2 ± 17,427.5 ^b^	73,100.8 ± 17,260.1 ^ab^	95,252.2 ± 175,789 ^a^	0.004
Aminopeptidase (RFU/mg protein)					
	10 DAH	n.d	n.d	n.d	
	24 DAH	n.d	n.d	n.d	
	31 DAH	2806.4 ± 280.9	2169.3 ± 594.3	2716.4 ± 940.9	0.463
4C-like lipase (RFU/mg protein)					
	10 DAH	15,523.4 ± 3479.0 ^ab^	18,834.0 ± 2671.4 ^a^	11,843.0 ± 3328.1^b^	0.001
	24 DAH	3638.0 ± 1109.1 ^a^	2852.2 ± 1268.9 ^b^	2726.1 ± 1031.6 ^b^	0.001
	31 DAH	4297.7 ± 610.7	4564.5 ± 1104.4	4842.7 ± 436.3	0.537
18C-like lipase (RFU/mg protein)					
	10 DAH	n.d	n.d	n.d	
	24 DAH	67,138.1 ± 11,514.5 ^a^	42,311.6 ± 15,716.9 ^b^	38,381.8 ± 9563.8 ^b^	<0.001
	31 DAH	26,624.9 ± 2486.3	25,552.0 ± 10,114.4	26,330.4 ± 5902.6	0.966
Alk phosphatase (RFU/mg protein)					
	10 DAH	n.d	n.d	n.d	
	24 DAH	156,951.4 ± 56,199.1 ^a^	123,276.2 ± 43,477.0 ^b^	139,037.9 ± 55,263 ^ab^	0.016
	31 DAH	207,951.5 ± 36,001.9	237,011.1 ± 53,083.7	221,804.8 ± 72,089.3	0.705
Amylase (RFU/mg protein)					
	10 DAH	n.d	n.d	n.d	
	24 DAH	78,820.7 ± 38,379.5	782,09.6 ± 39,566.6	71,996.3 ± 27,531.5	0.637
	31 DAH	43,469.5 ± 17,852.6	28,195.8 ± 7710.2	37,535.2 ± 11,410.4	0.201

Values are expressed as mean ± SD. Different letters indicate statistical differences between dietary treatments at the same larval age (a, b; *p* < 0.05, 1-way ANOVA). Absence of letters indicates no statistical differences (*p* > 0.05). Alk phosphatase, alkaline phosphatase; DAH, days after hatching; n.d, not detected.

**Table 4:** Biomarkers of oxidative status in gilthead seabream larvae throughout ontogeny fed different diets (CTRL, LOW, and HIGH).

		Larvae Age (Days after Hatching)			1-Way Anova
		10 DAH	24 DAH	31 DAH	*p* Value
GSH (μM/min/mg protein)					
	CTRL	8.1 ± 3.8 ^β^	68.6 ± 29.0 ^α^	68.3 ± 1.9 ^α^	0.007
	LOW	17.9 ± 8.1	68.6 ± 33.2	73.7 ± 5.5	0.134
	HIGH	12.9 ± 1.4 ^β^	69.1 ± 20.7 ^α^	67.1 ± 14.5 ^α^	0.022
TAC (mM Trolox equivalents/mg protein)					
	CTRL	687.1 ± 59.1 ^α^	497.2 ± 36.8 ^β^	560.3 ± 28.7 ^β^	0.005
	LOW	667.1 ± 63.3 ^α^	512.2 ± 41.8 ^β^	534.4 ± 57.8 ^αβ^	0.027
	HIGH	681.3 ± 63.2	539.6 ± 32.9	620.2 ± 61.8	0.051
PC (nmol carbonyl/mg protein)					
	CTRL	27.8 ± 6.9	33.1 ± 15.0	40.6 ± 36.2	0.924
	LOW	52.9 ± 10.7	51.8 ± 29.1	58.6 ± 24.3	0.927
	HIGH	23.7 ± 17.5	70.2 ± 23.6	26.4 ± 13.5	0.058
mtROS (RFU/mg protein)					
	CTRL	23.2 ± 16.1 ^β^	172.7 ± 17.8 ^α^	144.0 ± 25.7 ^α^	0.000
	LOW	29.7 ± 7.6	116.9 ± 66.4	81.0 ± 16.8	0.092
	HIGH	34.8 ± 6.2	98.5 ± 21.0	119.7 ± 65.2	0.065

Values are expressed as mean ± SD. Different letters indicate statistical differences between larvae ages in the same dietary treatment (α, β; *p* < 0.05, 1-way ANOVA). Absence of letters indicates no statistical differences (*p* > 0.05). GSH, glutathione; TAC, total antioxidant capacity; PC, protein carbonyl; mtROS, mitochondrial reactive oxygen species.

## Data Availability

The dataset supporting this study are present within the article.
